# Platelet-Rich Plasma Ameliorates Cyclophosphamide-Induced Acute Interstitial Cystitis/Painful Bladder Syndrome in a Rat Model

**DOI:** 10.3390/diagnostics10060381

**Published:** 2020-06-08

**Authors:** Yung-Hsiang Chen, Kee-Ming Man, Wen-Chi Chen, Po-Len Liu, Kao-Sung Tsai, Ming-Yen Tsai, Yu-Tzu Wu, Huey-Yi Chen

**Affiliations:** 1 Graduate Institute of Integrated Medicine, College of Chinese Medicine, China Medical University, Taichung 40402, Taiwan; yhchen@mail.cmu.edu.tw (Y.-H.C.); wgchen@mail.cmu.edu.tw (W.-C.C.); 2 Departments of Medical Research, Urology and Obstetrics and Gynecology, China Medical University Hospital, Taichung 40447, Taiwan; 3 Department of Psychology, College of Medical and Health Science, Asia University, Taichung 41354, Taiwan; 4 Department of Life Sciences, National Chung Hsing University, Taichung 40227, Taiwan; man_jimmy60@hotmail.com; 5 Department of Anesthesiology, China Medical University Hsinchu Hospital, Hsinchu 30272, Taiwan; 6 Department of Medicinal Botanicals and Health Applications, Da Yeh University, Changhua 51591, Taiwan; 7 Department of Respiratory Therapy, College of Medicine, Kaohsiung Medical University, Kaohsiung 80708, Taiwan; kisa@kmu.edu.tw; 8 Department of Applied Cosmetology, Hungkuang University, Taichung 43302, Taiwan; raysclinic@gmail.com; 9 Department of Chinese Medicine, Kaohsiung Chang Gung Memorial Hospital and Chang Gung University College of Medicine, Kaohsiung 83301, Taiwan; missuriae0116@gmail.com; 10 Department of Neurology, Kuang Tien General Hospital, Taichung 43303, Taiwan; inya15@yahoo.com

**Keywords:** platelet-rich plasma, interstitial cystitis, painful bladder syndrome, cyclophosphamide

## Abstract

Background: Interstitial cystitis/painful bladder syndrome (IC/PBS) could be treated to ameliorate urothelial injury. Here, we investigated the efficacy of intravesical instillation with platelet-rich plasma (PRP) and hyaluronic acid for acute IC/PBS. Methods: The effects of PRP and hyaluronic acid on the proliferation of normal human fibroblast cells (HFCs) were assessed. Additionally, thirty virgin female rats were randomized into five groups: group 1, saline-injected control; group 2, cyclophosphamide (CYP) plus intravesical instillation with normal saline; group 3, CYP plus intravesical instillation with hyaluronic acid (1 mg/mL); group 4, CYP plus intravesical instillation with PRP; and group 5, CYP plus intravesical instillation with PRP plus hyaluronic acid. A cystometry and histological assessments were performed. The expression of cell junction-associated protein zonula occludens-2 (ZO-2) and inflammatory cytokine interleukin 6 (IL-6) was also measured. Results: Low dose PRP increased proliferation in HFCs. The acute IC/PBS rats showed significantly lower voiding interval values. Voiding interval values were significantly higher in the CYP plus intravesical instillation with PRP group than in the CYP-induced acute IC/PBS group. Additionally, the expression of ZO-2 was increased and IL-6 was decreased in the CYP plus intravesical instillation with PRP group compared with the CYP-induced acute IC/PBS group. Conclusion: These findings suggest that PRP modulate urothelial repair, which ameliorate the increase in urination frequency in rats treated with CYP. Overall, PRP may confer potential benefits by acting as urothelial repair modulators.

## 1. Introduction

Interstitial cystitis/painful bladder syndrome (IC/PBS) is a chronic illness regarded as clinical by recurring events of pelvic pain and increased urination frequency [1,2], significantly damaging patients’ quality of life. Studies have stated the incidence of IC/PBS to be as high as 52–67 per 100,000 cases in the United States [3] and previous reports proposed that its prevalence is estimated to be in the range of 45 per 100,000 adult female population [4,5]. Due to the absence of certain diagnostic criteria and diversity of clinical presentation, IC/PBS is a very hard condition to treat. The inefficiency of IC/PBS therapies in some way at least results from a poor comprehension of the etiology and pathogenesis of the syndrome. In light of the limited availability of human tissue for study, animal models are an imperative adjunct in improving our comprehension of IC/PBS.

Based on clinical surveillances, bladder inflammation caused by a single intraperitoneal injection of cyclophosphamide (CYP) in rodents is a commonly used noninvasive model of acute IC/PBS [6,7]. Interleukin 6 (IL-6) is a proinflammatory cytokine that is generated by many types of cells and is expressed during states of cellular stress, such as inflammation, infection, wound sites, and cancer [8]. Many typical appearances of IC/PBS, such as pelvic pain, frequent urination, and activation of inflammation, emerge within 24 hours after injection. Though, this model includes dramatic alterations in bladder tissue morphology. Marked edema, huge inflammatory cell infiltration, tissue hemorrhages, and mucosal ulcerations [9,10] make this model more related to the hemorrhagic cystitis and ulcerative type of IC/PBS, which has a <30% prevalence in patients with IC/PBS [11]. While IC/PBS is a chronic pain disorder, the usefulness of this model in general, and especially to acute studies, is limited. The most putative theory on the pathogenesis of IC/PBS is affront to the epithelial glycosaminoglycan layer and loss of urothelium integrity, which causes potassium leakage into the interstitium [12]. Urothelial cell junctions perform an important role in the formation of the blood–urine barrier. Rickard et al. presented that the tight junction-associated zonula occludens proteins (ZO-1, ZO-2, and ZO-3) were localized in cell margins [13]. For example, the damage to the urothelial tight junctions disturbs barrier function, which then leads to urinary tract diseases.

Platelet-rich plasma (PRP) is leukocyte-free autologous plasma, mixed with plasma proteins produced from the patient’s own blood. It includes factors, such as platelet-derived growth factor, transforming growth factor, insulin-like growth factor, and epidermal growth factor, which initiate wound healing and tissue repair; as well as plasma proteins, such as fibrin, fibronectin, and vitronectin, which conduce to hemostasis and adhesion [14]. The factors in PRP facilitate and form healing, cellular mitogenesis, chemotaxis, differentiation, and metabolism [15]. Within the last two decades, PRP has received outstanding interest from medical specialties, including sports medicine, orthopedics, and maxillofacial surgery because of its wound-healing effects [16]. Lately, intravesical injections of PRP were reported to be safe and effective to increase bladder capacity and offer greater pain relief in patients with IC/PBS [17]. Intravesical instillation using hyaluronic acid also safely decreased the pain and, to a lesser degree, the urination frequency associated with IC/PBS [18]. Recent clinical studies and meta-analysis also showed that intravesical hyaluronic acid instillation improved quality of life, pain symptoms, and various outcomes, and could be included as therapeutic modality of IC/PBS [19,20,21].

Based on previous studies, PRP or hyaluronic acid could improve pelvic pain and urination frequency, which are involved in a wide variety of bladder dysfunction after urothelial injury, such as inflammation, overactive bladder, and IC/PBS [22]. Despite all this evidence, the full biological reasons of PRP and hyaluronic acid have not yet been clearly understood. Therefore, we hypothesized that this disease could be treated to ameliorate urothelial injury. In this study, the efficacy of intravesical instillation with PRP and hyaluronic acid for acute IC/PBS was investigated.

## 2. Materials and Methods

### 2.1. Ethical Statement

The processes of the animal study accepted consent from the ethical committee of the Institutional Animal Care and Use Committee of China Medical University (Reference number: CMUIACUC-2018-162, 27 December 2017).

### 2.2. Preparation of PRP

PRP was prepared as reported by Nagae et al. at room temperature [23]. Under sedation, the intracardiac route was used to remove blood because the filling rate for superficial veins would have been very slow and coagulation would have begun instantly during filling. A mean of 4.5 mL (3.5–6.0 mL) of blood was immediately injected into 4.5 mL tubes containing 3.2% citrate. Blood samples were received prior to the instillation and were instantly sent to the laboratory to prepare the PRP. The samples were centrifuged at 1600 rpm for 15 min to attain separation of the supernatant and buffy coat. The remaining plasma was then centrifuged at 2800 rpm for another 8 minutes and the thrombocytes were precipitated. The supernatant, which contained fewer platelets, was thrown away and a mean 1 mL (0.8–1.2 mL) of PRP was acquired from the buffy coat. Freshly prepared 0.3 mL of PRP was given intravesically to the PRP groups. PRP was left in the bladder for 1 h duration. A sterile 18-gauge angiocath was inserted into the bladder through the urethral opening with 1 mL syringe [24].

### 2.3. Cell Culture and 3-(4,5-dimethylthiazol-2-yl)-2,5-diphenyl Tetrazoliumbromide (MTT) Assay for Cell Proliferation

Normal human skin fibroblast cells (HFCs) (Name: CG1476, BCRC Number: 08C0057, Bioresource Collection and Research Center, Hsinchu, Taiwan) were grown in culture medium (85% Dulbecco’s modified Eagle’s medium (DMEM) with 4 mM L-glutamine adjusted to contain 1.5 g/L sodium bicarbonate and 4.5 g/L glucose + 15% fetal bovine serum). HFCs were grown in 96 well culture plates at a concentration of 3 × 10^4^ cells/well for 24 h and then serum-free medium with various concentrations of PRP was added. The cells were collected 24 h later. MTT (0.5 mg/mL) was applied to the cells for 4 h to allow the conversion of MTT into formazan crystals, the cells were lysed with dimethyl sulfoxide (DMSO), and the absorbance was read at 570 and 650 nm with a DIAS Microplate Reader (Molecular Devices, Sunnyvale, CA, USA). The reduction in optical density caused by treatment was used as a measurement of cell proliferation, normalized to the cells incubated in control medium, which were considered 100% viable [25].

### 2.4. Animal Model and Cystometry

**Figure 1:**
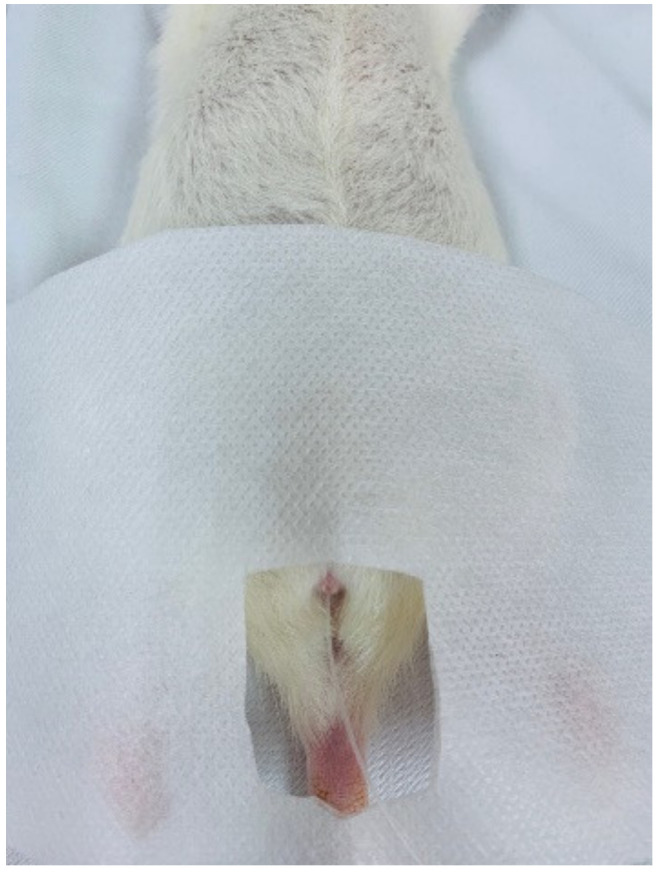
A represented experimental photo for the analysis of cystometry.

Thirty female virgin rats (Sprague Dawley; 225–250 g), aged 6–8 weeks, were randomized into five groups (*n* = 6/each group): group 1, saline-injected control; group 2, CYP plus intravesical instillation with normal saline; group 3, CYP plus intravesical instillation with hyaluronic acid (1 mg/mL); group 4, CYP plus intravesical instillation with PRP (0.3 mL); and group 5, CYP plus intravesical instillation with PRP plus hyaluronic acid. First, CYP-induced urothelial injury was provoked by a single intraperitoneal (i.p.) injection of CYP at a dose of 150 mg/kg dissolved in normal saline (5 mL/kg) [8,9,10]. Control rats received saline injections. Injections were done on day 0. Rats received intravesical instillation with hyaluronic acid, PRP, or normal saline on day 1. Experiments were conducted on day 4. The procedure followed urethane (1 g/kg, i.p.) anesthesia, in line with the methods as aforementioned. A sterile PE-10 catheter was inserted in the bladder through the urethra and an intravesical administration of saline (Figure 1) [26,27]. The sterile bladder catheter was linked to a syringe pump and a pressure transducer recorded pressure. Pressure and force transducer signals were examined and interpreted from data collection at 10 samples per second (PowerLabs, ADInstruments, Bella Vista, Australia). The rats were put supine, the pressure was at the level of zero, and bladders were filled with room temperature saline (20 μL/min) through the sterile bladder catheter at the same time. After a 30 min equilibration period, intravesical pressure was collected for 30 min [28].

### 2.5. Histological Examination

The rats were sacrificed directly after completing cystometry and the bladders were reaped. The tissue samples were fixed in 10% buffered formalin (pH 7.4) and applied for hematoxylin and eosin stain (HE stain). The specimens were studied using light microscopy and photographed.

### 2.6. Protein Preparation and Western Blot Analysis

Frozen tissue samples were ground with a liquid nitrogen-chilled mortar and pestle. Tissue powder was then homogenized in buffer (16 mM potassium phosphate, pH 7.8, 0.12 mol/L NaCl, 1 mM ethylenediaminetetraacetic acid) including a protease inhibitor cocktail (Complete Mini, Roche Diagnostics, Mannheim, Germany), and then centrifuged at 10,000 *g*. The supernatant was removed and the previous homogenization step was done again after resuspending the remaining tissue pellet in basic buffer. After the removal of the second supernatant, the remaining tissue pellet was suspended in urea buffer (6 M). The samples were centrifuged (13,000× *g* for 30 min) and the supernatant was discarded. Protein concentration was defined using bicinchoninic acid protein assay (Pierce, Rockford, IL, USA). Protein samples are analyzed by proper percentage of polyacrylamide gel electrophoresis. Later, proteins in the gel were transferred onto the polyvinylidene difluoride (PVDF) membrane with a semi-dry transfer unit at 0.8 mA/cm^2^ for 2 h. The PVDF blots were blocked in Tris Buffered Saline Buffer with Tween 20 (TBST) containing 2% non-fat dry milk, hatched with a primary antibody, followed by a secondary antibody conjugated with horseradish peroxidase. Proteins of interest were visualized with an ECL system, followed by autoradiography.

### 2.7. Statistical Analysis

Quantitative data are expressed as the mean ± standard deviation (SD). Since a small number of samples did not conform to the normally distributed continuous variables, nonparametric methods of Mann-Whitney U test and Kruskal-Wallis test inference were used. *p*-value less than 0.05 was considered statistically significant. All calculations were performed using the Statistical Package for Social Sciences (SPSS for Windows, release 8.0, SPSS Inc., Chicago, IL, USA).

## 3. Results

### 3.1. Effects of PRP on Cell Proliferation of HFCs

**Figure 2:**
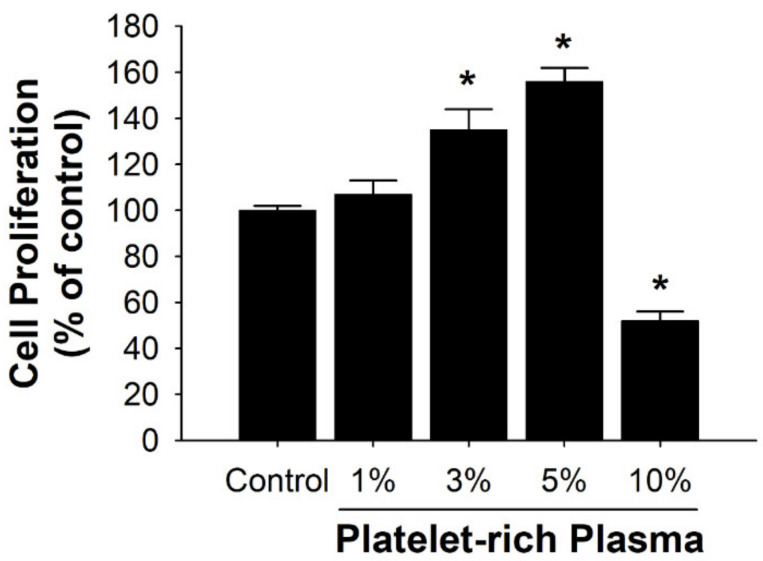
Effects in different concentrations of platelet-rich plasma (PRP) on cell proliferation of human fibroblast cells (HFCs) evaluated by MTT assays. Data are presented as the mean ± SD of three experiments performed in triplicate. * *p* < 0.05 contrasted to the control group.

The effects of PRP on the human HFCs proliferation were surveyed. HFCs were treated with various concentrations of PRP (1–10%) for 24 h and cell proliferation was quantified by MTT assay. MTT assays disclosed a dose-dependent (1–5%) increase in mitochondrial succinate dehydrogenase activity in the HFCs following exposure to PRP. However, the highest concentration (10%) caused a significant reduction in cell proliferation (Figure 2) (*p* < 0.05, contrasted to control group).

### 3.2. Bladder Effect of PRP in a Rat Model of CYP-induced Acute IC/PBS

**Figure 3:**
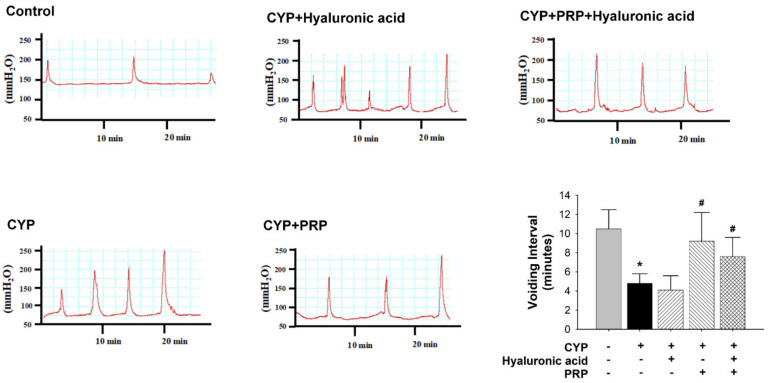
Cystometry traces between saline-injected control, cyclophosphamide (CYP) plus intravesical instillation with normal saline, CYP plus intravesical instillation with hyaluronic acid (1 mg/mL), CYP plus intravesical instillation with PRP, and CYP plus intravesical instillation with hyaluronic acid (1 mg/mL) plus PRP groups. The peaks in the cystometry indicate the detrusor contraction (overactivity was shown in each group). Voiding interval values are revealed in the different groups. Each bar represents the mean ± SD of six individual rats. * *p* < 0.05 compared to the control group. # *p* < 0.05 contrasted to the CYP group.

Contrasted to those in the control group, the voiding interval values (time between voids) in the CYP plus intravesical instillation with normal saline and hyaluronic acid (1 mg/mL) groups significantly decreased. Additionally, contrasted to those in the CYP-induced acute IC/PBS group, the voiding interval values in the CYP plus intravesical instillation with PRP group (but not CYP plus hyaluronic acid and CYP plus PRP plus hyaluronic acid groups) significantly increased (Figure 3).

**Figure 4:**
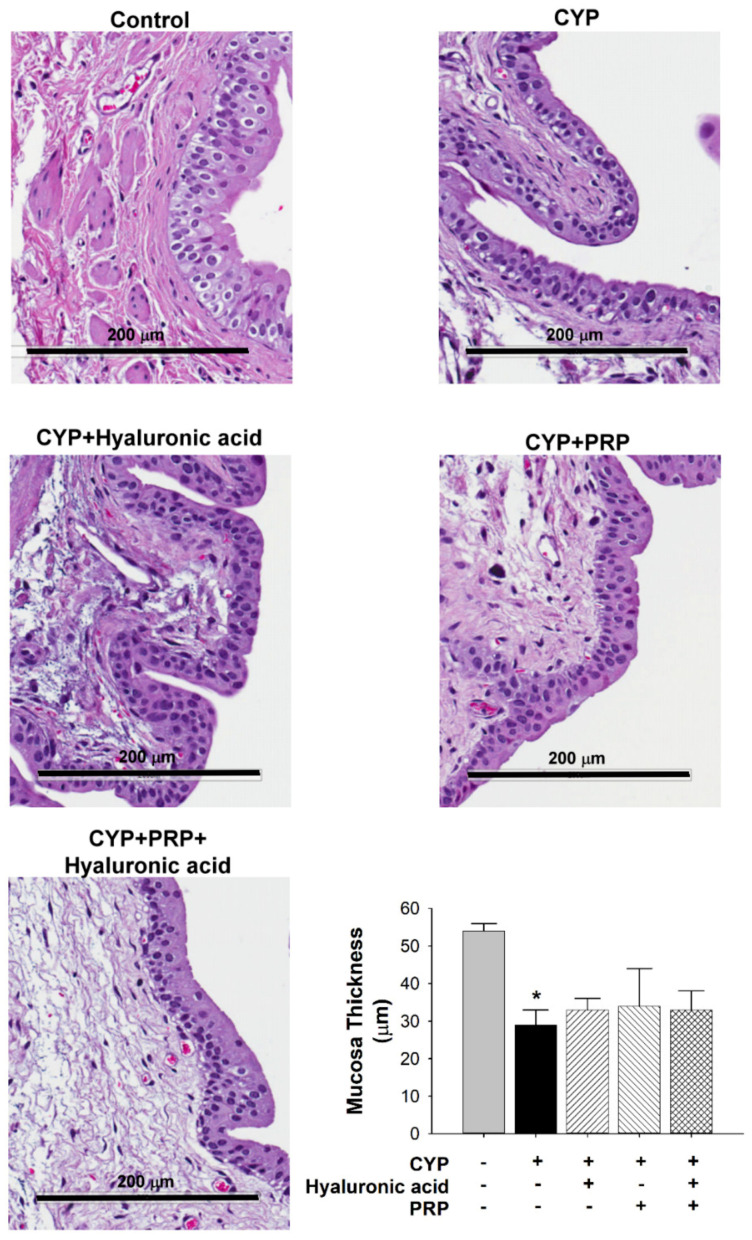
Average thickness of mucosa between saline-injected control; CYP plus intravesical instillation with normal saline; CYP plus intravesical instillation with hyaluronic acid (1 mg/mL); CYP plus intravesical instillation with PRP; and CYP plus intravesical instillation with hyaluronic acid (1 mg/mL) plus PRP groups. Each bar denotes the mean ± SD of six individual rats. * *p* < 0.05 contrasted to the control group.

**Figure 5:**
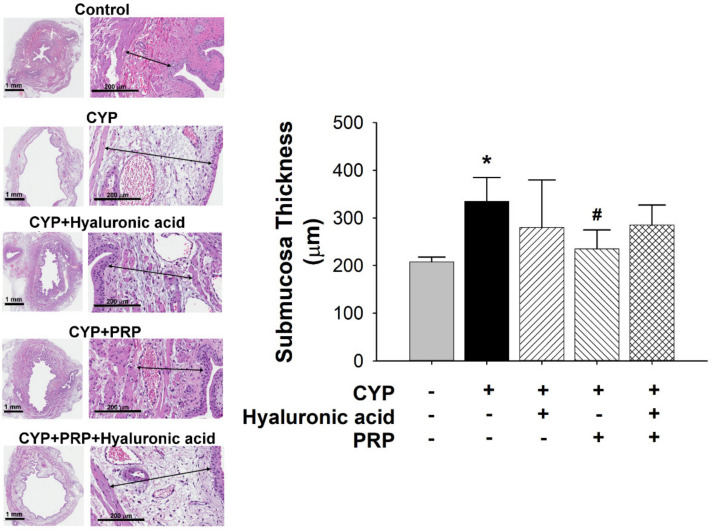
Average thickness of submucosa between saline-injected control, CYP plus intravesical instillation with normal saline, CYP plus intravesical instillation with hyaluronic acid (1 mg/mL), CYP plus intravesical instillation with PRP, and CYP plus intravesical instillation with hyaluronic acid (1 mg/mL) plus PRP groups. Each bar denotes the mean ± SD of six individual rats. * *p* < 0.05 contrasted to the control group. # *p* < 0.05 compared to the CYP group.

Normal urothelium is normally comprised of transitional epithelium but it seems that the most superficial cell layer was destroyed after CYP exposure (Figure 4). By contrast, submucosal thickness in the bladder of CYP group was significantly increased as compared to control. The submucosal thickness in the bladder of CYP plus PRP group was significantly decreased as contrasted to CYP group (Figure 5).

### 3.3. ZO-2 and IL-6 Expression in a Rat Model of CYP-induced Acute IC/PBS

**Figure 6:**
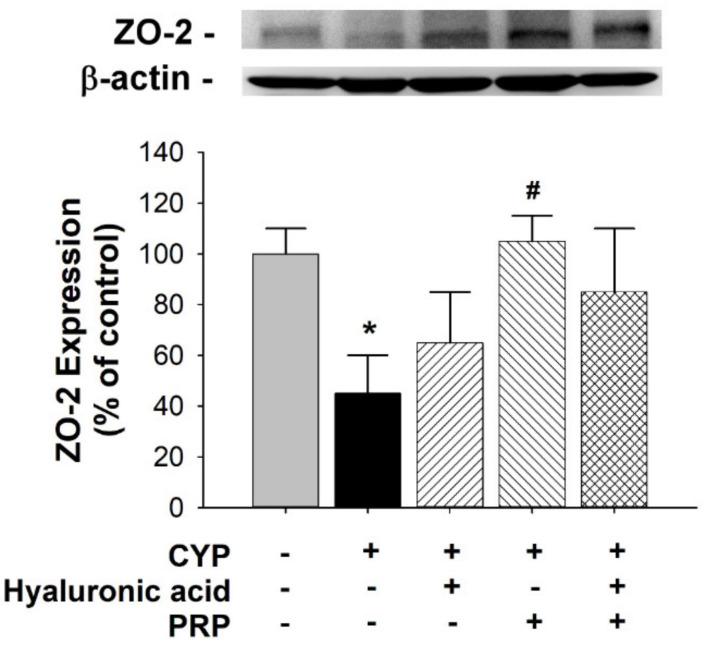
Alteration of ZO-2 expression in the bladder as indicated by Western blot analyses between saline-injected control, CYP plus intravesical instillation with normal saline, CYP plus intravesical instillation with hyaluronic acid (1 mg/mL), CYP plus intravesical instillation with PRP, and CYP plus intravesical instillation with hyaluronic acid (1 mg/mL) plus PRP groups. Each bar denotes the mean ± SD of six individual rats. * *p* < 0.05 compared to the control group. # *p* < 0.05 contrasted to the CYP group.

**Figure 7:**
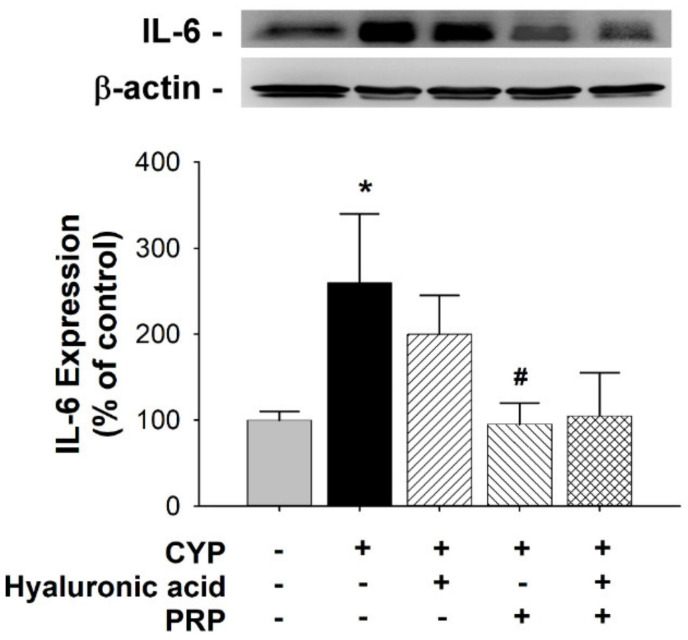
Alteration of IL-6 expression in bladder as indicated by Western blot analyses between saline-injected control, CYP plus intravesical instillation with normal saline, CYP plus intravesical instillation with hyaluronic acid (1 mg/mL), CYP plus intravesical instillation with PRP, and CYP plus intravesical instillation with hyaluronic acid (1 mg/mL) plus PRP groups. Each bar denotes the mean ± SD of six individual rats. * *p* < 0.05 contrasted to the control group. # *p* < 0.05 compared to the CYP group.

The tight junction-associated zonula occludens protein ZO-2 was localized in cell margins for barrier function. Therefore, we examined the ZO-2 expression for urothelial integrity. Compared to that in the control group, ZO-2 expression significantly decreased in the bladders of the CYP group. By contrast, the ZO-2 expression in the CYP plus PRP group was significantly increased as compared to CYP group (Figure 6). Interleukin 6 (IL-6) is a proinflammatory cytokine. Therefore, we analyzed the IL-6 expression for urothelial injury. Compared to that in the control group, IL-6 expression significantly increased in the bladders of the CYP group. By contrast, the IL-6 expression in the CYP plus PRP group was significantly decreased as compared to CYP group (Figure 7).

## 4. Discussion

The urothelium, a specialized lining of the urinary tract, has historically been said as a high-resistance barrier to ions, solutes, water flux, and pathogens. Urothelium is comprised of at least three layers: a basal cell layer attached to a basement membrane, an intermediate layer, and a superficial or apical layer composed of large hexagonal cells (diameters of 25–250 μm) identified as “umbrella cells” [29,30]. Characteristically, the urothelium has a turnover rate of about 3–6 months.

The urothelial barrier function is founded on the physiological regeneration or pathological repair of the urothelium. In this study, low doses of PRP increased cell proliferation in HFCs contrasted to the untreated control, indicating enhanced cell proliferation with PRP. Our data suggest that increased proliferation of the urothelium could be imputed to activation by PRP.

Urothelial injury caused by various noxious agents (chemical, physical, infectious, and inflammatory) may lead to increased penetration and absorption of toxic substances, immunogenic responses, and disequilibrium of the host’s homeostasis. The toxic substances can pass into the underlying tissue (neural and/or muscle layers), which influence urination frequency and urgency and lead to bladder discomfort or pain. These symptoms are involved in an extensive variety of bladder dysfunction, such as inflammation, overactive bladder, and IC/PBS [22].

In the present study, we observed that urothelial thickness decreased in the bladder of rats with acute IC/PBS. These findings suggested that damage to the urothelium might lead to typical signs of a leaky barrier, which may result in an increased urination frequency in acute IC/PBS rats. Voiding interval values were significantly increased in the CYP plus intravesical instillation with PRP group. These findings suggest that PRP modulated urothelial repair, which ameliorated the increased urination frequency in rats with acute IC/PBS. Furthermore, ZO-2 expression in the bladder increased in the CYP plus intravesical instillation with PRP group, and IL-6 expression in the bladder decreased in the CYP plus intravesical instillation with PRP group. Our data suggest that ZO-2 expression can be upregulated by PRP and IL-6 expression can be downregulated by PRP. There was no synergistic effect when PRP and hyaluronic acid were given together. PRP seems to be superior to hyaluronic acid in the urothelial repair. There is evidence that PRP may perform an important role in urothelial repair.

Our study has certain limitations: (1) the pelvic floor structure of the rat, which is a quadruped and has a lax abdominal wall, is different from that of a human female [31]; therefore, the results of this study need to be carefully employed to humans; (2) urodynamic studies were conducted under anesthesia [32]; nevertheless, the preoperative fasting water was not restricted; (3) the effects of PRP on proliferation, maturation, and differentiation of undifferentiated urothelial cells were not observed separately in vitro in the present study; (4) this rat model generated by a single intraperitoneal injection of CYP is an acute IC/PBS model, while human IC/PBS is a chronic disease. It may be the case that several models, each contributing to a piece of the puzzle, are required to recreate a reasonable picture of the pathophysiology and time course of the disease(s) diagnosed as IC/PBS, and thus to identify reasonable targets for treatment [29]. In addition, PRP was prepared at room temperature in this study, however, many authors used a temperature level of 12 °C–16 °C during centrifugation to better obtain PRP because cooling can retard platelet activation and this is essential in obtaining PRP with viable platelets [33]. More studies to translate these findings into new therapeutics for IC/PBS women are ensured for further clarity.

## 5. Conclusions

Our results suggest that CYP can induce acute IC/PBS in female rats. Intravesical instillation with PRP in CYP-treated rats can help resolve acute IC/PBS. PRP accelerated urothelial repair by stimulating cell proliferation, ZO-2 expression, or downregulating IL-6 expression. PRP mediated the increase in bladder function in rats with acute IC/PBS rats via downregulation of IL-6 expression and kept urothelial integrity via upregulation of ZO-2 expression, suggesting the important role of urothelial repair in IC/PBS. Overall, PRP may confer potential benefits as urothelial repair modulators.

## References

[1] Batista R., Vinagre N., Meireles S., Vinagre J., Prazeres H., Leao R., Maximo V., Soares P. (2020). Biomarkers for Bladder Cancer Diagnosis and Surveillance: A Comprehensive Review. Diagnostics.

[2] Akiyama Y., Luo Y., Hanno P.M., Maeda D., Homma Y. (2020). Interstitial cystitis/bladder pain syndrome: The evolving landscape, animal models and future perspectives. Int. J. Urol..

[3] Rahnama’i M.S., Marcelissen T., Apostolidis A., Veit-Rubin N., Schurch B., Cardozo L., Dmochowski R. (2018). The efficacy of botulinum toxin A and sacral neuromodulation in the management of interstitial cystitis (IC)/bladder pain syndrome (BPS), what do we know? ICI-RS 2017 think thank, Bristol. Neurourol. Urodyn..

[4] Patnaik S.S., Lagana A.S., Vitale S.G., Buttice S., Noventa M., Gizzo S., Valenti G., Rapisarda A.M.C., La Rosa V.L., Magno C. (2017). Etiology, pathophysiology and biomarkers of interstitial cystitis/painful bladder syndrome. Arch. Gynecol. Obstet..

[5] Meng E., Hsu Y.C., Chuang Y.C. (2018). Advances in intravesical therapy for bladder pain syndrome (BPS)/interstitial cystitis (IC). Low Urin Tract Symptoms.

[6] Auge C., Chene G., Dubourdeau M., Desoubzdanne D., Corman B., Palea S., Lluel P., Vergnolle N., Coelho A.M. (2013). Relevance of the cyclophosphamide-induced cystitis model for pharmacological studies targeting inflammation and pain of the bladder. Eur. J. Pharmacol..

[7] Boucher M., Meen M., Codron J.P., Coudore F., Kemeny J.L., Eschalier A. (2000). Cyclophosphamide-induced cystitis in freely-moving conscious rats: Behavioral approach to a new model of visceral pain. J. Urol..

[8] Felsen D., Frye S., Vaughan E.D. (1991). Inflammatory mediators and interstitial cystitis. Semin. Urol..

[9] Juszczak K., Gil K., Wyczolkowski M., Thor P.J. (2010). Functional, histological structure and mastocytes alterations in rat urinary bladders following acute and [corrected] chronic cyclophosphamide treatment. J. Physiol. Pharmacol..

[10] Smaldone M.C., Vodovotz Y., Tyagi V., Barclay D., Philips B.J., Yoshimura N., Chancellor M.B., Tyagi P. (2009). Multiplex analysis of urinary cytokine levels in rat model of cyclophosphamide-induced cystitis. Urology.

[11] Simon L.J., Landis J.R., Erickson D.R., Nyberg L.M. (1997). The Interstitial Cystitis Data Base Study: Concepts and preliminary baseline descriptive statistics. Urology.

[12] Cervigni M. (2015). Interstitial cystitis/bladder pain syndrome and glycosaminoglycans replacement therapy. Transl. Androl. Urol..

[13] Rickard A., Dorokhov N., Ryerse J., Klumpp D.J., McHowat J. (2008). Characterization of tight junction proteins in cultured human urothelial cells. In Vitro Cell. Dev. Biol. Anim..

[14] Donmez M.I., Inci K., Zeybek N.D., Dogan H.S., Ergen A. (2016). The Early Histological Effects of Intravesical Instillation of Platelet-Rich Plasma in Cystitis Models. Int. Neurourol. J..

[15] Garg A.K. (2000). The use of platelet-rich plasma to enhance the success of bone grafts around dental implants. Dent. Implantol. Update.

[16] Marx R.E. (2004). Platelet-rich plasma: Evidence to support its use. J. Oral Maxillofac. Surg..

[17] Jhang J.F., Lin T.Y., Kuo H.C. (2019). Intravesical injections of platelet-rich plasma is effective and safe in treatment of interstitial cystitis refractory to conventional treatment—A prospective clinical trial. Neurourol. Urodyn..

[18] Kallestrup E.B., Jorgensen S.S., Nordling J., Hald T. (2005). Treatment of interstitial cystitis with Cystistat: A hyaluronic acid product. Scand. J. Urol. Nephrol..

[19] Gulpinar O., Esen B., Kayis A., Gokce M.I., Suer E. (2018). Clinical comparison of intravesical hyaluronic acid and chondroitin sulfate therapies in the treatment of bladder pain syndrome/interstitial cystitis. Neurourol. Urodyn..

[20] Liang C.C., Lin Y.H., Hsieh W.C., Huang L. (2018). Urinary and psychological outcomes in women with interstitial cystitis/bladder pain syndrome following hyaluronic acid treatment. Taiwan J. Obstet. Gynecol..

[21] Pyo J.S., Cho W.J. (2016). Systematic Review and Meta-Analysis of Intravesical Hyaluronic Acid and Hyaluronic Acid/Chondroitin Sulfate Instillation for Interstitial Cystitis/Painful Bladder Syndrome. Cell Physiol. Biochem..

[22] Keay S.K., Birder L.A., Chai T.C. (2014). Evidence for bladder urothelial pathophysiology in functional bladder disorders. Biomed. Res. Int..

[23] Nagae M., Ikeda T., Mikami Y., Hase H., Ozawa H., Matsuda K., Sakamoto H., Tabata Y., Kawata M., Kubo T. (2007). Intervertebral disc regeneration using platelet-rich plasma and biodegradable gelatin hydrogel microspheres. Tissue Eng..

[24] Ozyuvali E., Yildirim M.E., Yaman T., Kosem B., Atli O., Cimentepe E. (2016). Protective Effect of Intravesical Platelet-Rich Plasma on Cyclophosphamide-Induced Hemorrhagic Cystitis. Clin. Investig. Med..

[25] Chen Y.H., Chen W.C., Liu P.L., Chen H.Y. (2020). Astragalus polysaccharides and astragaloside IV ameliorates cyclophosphamide-induced mouse model of overactive bladder. Taiwan J. Obstet. Gynecol..

[26] Monjotin N., Farrie M., Vergnolle N., Le Grand B., Gillespie J., Junquero D. (2017). Bladder telemetry: A new approach to evaluate micturition behavior under physiological and inflammatory conditions. Neurourol. Urodyn..

[27] Wang H.J., Lee W.C., Tyagi P., Huang C.C., Chuang Y.C. (2017). Effects of low energy shock wave therapy on inflammatory moleculars, bladder pain, and bladder function in a rat cystitis model. Neurourol. Urodyn..

[28] Boudes M., Uvin P., Kerselaers S., Vennekens R., Voets T., De Ridder D. (2011). Functional characterization of a chronic cyclophosphamide-induced overactive bladder model in mice. Neurourol. Urodyn..

[29] Apodaca G. (2004). The uroepithelium: Not just a passive barrier. Traffic.

[30] Lewis S.A. (2000). Everything you wanted to know about the bladder epithelium but were afraid to ask. Am. J. Physiol. Renal Physiol..

[31] Fujimura M., Izumimoto N., Momen S., Yoshikawa S., Kobayashi R., Kanie S., Hirakata M., Komagata T., Okanishi S., Hashimoto T. (2014). Characteristics of TRK-130 (Naltalimide), a novel opioid ligand, as a new therapeutic agent for overactive bladder. J. Pharmacol. Exp. Ther..

[32] Schneider M.P., Hughes F.M., Engmann A.K., Purves J.T., Kasper H., Tedaldi M., Spruill L.S., Gullo M., Schwab M.E., Kessler T.M. (2015). A novel urodynamic model for lower urinary tract assessment in awake rats. BJU Int..

[33] Dhurat R., Sukesh M. (2014). Principles and Methods of Preparation of Platelet-Rich Plasma: A Review and Author’s Perspective. J. Cutan. Aesthet. Surg..

